# Treatment with IMM-101 induces protective CD8+ T cell responses in clinically relevant models of pancreatic cancer

**DOI:** 10.1186/2051-1426-1-S1-P215

**Published:** 2013-11-07

**Authors:** Androulla Elia, Louise Lincoln, Laura Rosa Brunet, Thorsten Hagemann

**Affiliations:** 1 St George's, University of London, London, UK; 2 Barts Cancer Institute, QMUL, London, UK; 3 Immodulon Therapeutics Ltd, London, UK

## 

Pancreatic cancer is an aggressive cancer with poor prognosis. Despite its low incidence, it is the 4th cause of cancer-related death in the US. Treatment options have only marginally improved on survival rates, which have remained low, with about 25% survival at 12 months and 5% at 5 years. For these reasons, new therapeutic strategies are urgently needed including immunotherapeutic approaches. We have investigated the immunotherapeutic effect of IMM-101, a heat-killed whole cell preparation of Mycobacterium obuense currently undergoing investigation in a Phase II clinical trial in pancreatic cancer (EudraCT n. 2010-022757-42), in two clinically relevant murine models of pancreatic cancer, which histologically mirror human pancreatic adenocarcinomas. Genetically-modified mice bearing mutations in Kras, p53 and Pdx-Cre (KPC mice) were treated with IMM-101 immediately after development of a palpable tumour. Whereas IMM-101 treatment was unable to effect survival in this rather aggressive model, it did, however, significant decrease metastatic burden. Moreover, it appeared to expand a population of antigen experienced CD8+ T cells bearing CD45RBlowCD44high and able to produce IFN-γ and perforin. On the basis of these promising observations, we explored further whether treatment with IMM-101 could induce cytotoxic CD8+ T cells able to effect disease outcome. We treated mice bearing mutations in Kras and Pdx-Cre (KC mice) with IMM-101 and found that not only was survival significantly increased, but also that IMM-101 treatment altered their immune response to disease. We observed systemic T cell activation at the tumour site, the draining lymph nodes and the spleen, as measured by CD69 expression on T cells. More importantly, in mice treated with IMM-101, CD8+ T cells were found in higher numbers compared to untreated mice, in both the draining lymph nodes and at the tumour site. These CD8+ T cells were characterized by increased production of IFN-γ, perforin and granzyme, identifying them as cytotoxic CD8+ effector T cells. To further confirm that the mode of action of IMM-101 was directly depended on CD8+ T cells, we depleted these cells in treated mice with a neutralizing antibody. We found that depletion of CD8+ T cells, but not for example depletion of NK cells, was responsible for the loss of therapeutic effect. We are currently sequencing the CD8+ T cell TCR to determine specificity. We propose that treatment with IMM-101 is able to induce CD8+ T cell-dependent protective effects in the host and limit disease progression. We expect that in combination therapies these immunotherapeutic effects may be further increased.

